# Molecular Profiling of the *Phytophthora plurivora* Secretome: A Step towards Understanding the Cross-Talk between Plant Pathogenic Oomycetes and Their Hosts

**DOI:** 10.1371/journal.pone.0112317

**Published:** 2014-11-05

**Authors:** Valeria Severino, Annarita Farina, Frank Fleischmann, Ronaldo J. D. Dalio, Antimo Di Maro, Monica Scognamiglio, Antonio Fiorentino, Augusto Parente, Wolfgang Osswald, Angela Chambery

**Affiliations:** 1 Department of Environmental, Biological and Pharmaceutical Sciences and Technologies, Second University of Naples, Caserta, Italy; 2 Department of Human Protein Science, Geneva University, Geneva, Switzerland; 3 Section Pathology of Woody Plants, Technische Universität München (TUM), Freising-Weihenstephan, Germany; 4 IRCCS Multimedica, Milan, Italy; Seoul National University, Republic of Korea

## Abstract

The understanding of molecular mechanisms underlying host–pathogen interactions in plant diseases is of crucial importance to gain insights on different virulence strategies of pathogens and unravel their role in plant immunity. Among plant pathogens, *Phytophthora* species are eliciting a growing interest for their considerable economical and environmental impact. Plant infection by *Phytophthora* phytopathogens is a complex process coordinated by a plethora of extracellular signals secreted by both host plants and pathogens. The characterization of the repertoire of effectors secreted by oomycetes has become an active area of research for deciphering molecular mechanisms responsible for host plants colonization and infection. Putative secreted proteins by *Phytophthora* species have been catalogued by applying high-throughput genome-based strategies and bioinformatic approaches. However, a comprehensive analysis of the effective secretome profile of *Phytophthora* is still lacking. Here, we report the first large-scale profiling of *P. plurivora* secretome using a shotgun LC-MS/MS strategy. To gain insight on the molecular signals underlying the cross-talk between plant pathogenic oomycetes and their host plants, we also investigate the quantitative changes of secreted protein following interaction of *P. plurivora* with the root exudate of *Fagus sylvatica* which is highly susceptible to the root pathogen. We show that besides known effectors, the expression and/or secretion levels of cell-wall-degrading enzymes were altered following the interaction with the host plant root exudate. In addition, a characterization of the *F. sylvatica* root exudate was performed by NMR and amino acid analysis, allowing the identification of the main released low-molecular weight components, including organic acids and free amino acids. This study provides important insights for deciphering the extracellular network involved in the highly susceptible *P. plurivora*-*F. sylvatica* interaction.

## Introduction

During the last years, remarkable efforts have been focused on understanding the molecular mechanisms underlying host–pathogen interactions in plant diseases. Among plant pathogens, *Phytophthora* species are eliciting a growing interest for their considerable economical and environmental impact [1], [2]. These filamentous microorganisms are oomycetes, belonging to the Stramenopiles [3], that include many devastating pathogens causing severe plant diseases in agricultural plant communities and in natural ecosystems [4], [5]. In European forest, many *Phytopthora* species are correlated with the decline of different broad leaf trees. *P. plurivora* is one of the most frequently isolated *Phytophthora* species in middle European beech (*Fagus sylvatica*) and oak (*Quercus* spp.) forest [6]–[8].

Plant infection by *Phytophthora* phytopathogens is a complex process coordinated by a plethora of extracellular signals secreted by both host plants and pathogens [9]–[17]. In particular, *Phytophthora* species secrete many proteins that modulate plant innate immunity for infection. [9], [11], [18], [19] According to a definition introduced by Kamoun [11], these proteins, termed “effectors”, are molecules endowed with the ability to facilitate infection by altering host cell structure and function (virulence factors or toxins) in a host. Others were shown to trigger defense responses as avirulence factors, if the host carried corresponding resistance genes. Effectors can be targeted to the space outside plant cell membranes (apoplastic effectors) or translocated into the host cell (cytoplasmic effectors) [11].

It is even more evident that the knowledge of the repertoire of effector proteins secreted by oomycetes is essential for deciphering their biochemical activities and to understand molecular mechanisms responsible for host plants colonization and infection. Therefore, the characterization of molecules secreted by oomycetes has become an active area of research. Several studies reported the characterization of proteins released by *Phytophthora* species through genetic, biochemical and bioinformatic approaches [9]–[12]. In the genomic era, secreted proteins, traditionally isolated by biochemical purification, have been catalogued by applying high-throughput genome-based strategies. This approach allowed the generation of lists of putative secreted proteins (secretome) for a given *Phytophthora* species [11], [20]. In addition, the computational analysis of N-terminal secretion signal peptides also allowed the prediction of candidate secreted proteins by using bioinformatic tools [11], [21]. However, an intrinsic limitation of the *in silico* methodologies is that many secreted proteins that do not carry signal peptides cannot be identified using prediction algorithms.

Although several classes of apoplastic and cytoplasmic effectors have been identified or predicted, the array of secreted proteins involved in the host-pathogen interaction has not yet been fully elucidated. Indeed, a complex scenario is emerging on the secretome of pathogenic oomycetes with hundreds of proteins able to manipulate host functions [11]. In this context, a crucial aspect in the characterization of oomycete effectors is the analysis of the real proteins secreted in the extracellular space to reach their host plant cellular targets [21]. This challenging aim can be accomplished by directly analysing the protein complement secreted in *Phytophthora* culture filtrates by applying proteomics approaches. However, to date, studies on *Phytophthora* secretome by high-throughput -*omics* strategies are still in their infancy and have been mainly focused to investigate the plant defence response following pathogen infection [22]. In a pioneering study by Torto and coworkers, the identification of secreted proteins collected from culture filtrates of *P. infestans* was performed by two dimensional gel electrophoresis (2-DE) and MALDI-TOF MS, leading to the identification of twenty two proteins, nine of which were predicted to be secreted by the PexFinder algorithm [21].

To our knowledge, a comprehensive analysis of the secretome profile of *Phytophthora* is lacking.

Here, we thus report the first large-scale profiling of *P. plurivora* secretome using a shotgun LC-MS/MS strategy. To gain insight on the molecular signals underlying the cross-talk between plant pathogenic oomycetes and their host plants, we also investigate the quantitative changes of secreted protein following interaction of *P. plurivora* with the root exudate of *Fagus sylvatica* which is highly susceptible to the root pathogen.

Our results provide a detailed characterization of the *P. plurivora* secretome, revealing that, besides known effectors and potential pathogenicity factors, the expression and/or secretion levels of cell-wall-degrading enzymes were altered following the interaction with the host plant root exudate. In addition, a characterization of the *F. sylvatica* root exudate was performed by NMR and amino acid analysis, allowing the identification of the main released low-molecular weight components, including organic acids (i.e. formic acid, acetic acid, lactic acid and *p-*toluic acid) and free amino acids (e.g. pSer, Asp, Ser, Glu, Sar, Gly and Ala).

This study provides important insights for deciphering the complex extracellular network involved in the highly susceptible *P. plurivora*-*F. sylvatica* interaction.

## Materials and Methods

The field studies did not involve endangered or protected species. No specific permissions were required for these locations/activities.

### Composition of Henninger synthetic medium

One litre of Henninger synthetic medium [23] contains 0.4 g KH_2_PO_4_, 0.4 g NaNO_3_, 0.1 g CaCl_2_, 0.1 g MgCO_3_, 0.1 g (NH_4_)_2_SO_4_, 0.02 g FeSO_4_×7 H_2_O, 200 mg succinic acid, 200 mg arginine, 200 mg glycine, 400 mg aspartic acid, 400 mg glutamic acid, 100 mg alanine and 100 mg leucine, 150 mg cysteine HCl, 1 mg thiamine hydrochlorid, 10 g glucose and 5 g sucrose, pH 5.0.

### *Phytophthora plurivora* strain and culture conditions

*Phytophthora plurivora* T. Jung and T.I. Burgess, isolate CIT55, which was isolated from a declining beech in Southern Bavaria (Waldeck 6c, district XIV Kreuzjoch, Grainau, Germany; GPS coordinates: W/E 11.052; N/S 47.460; altitude 1030 m above sea level), was grown on V8 agar in the dark at 20°C. For culture filtrate preparation, *P. plurivora* was grown in 1.25 L of autoclaved (121°C, 30 min) Henninger liquid culture medium at 20°C with shaking at 120 rpm for 8 days [23]. The culture filtrates were then filtered on 0.2 µm filters and lyophilized. Samples stimulated with *F. sylvatica* root exudate were prepared by directly dissolving the salts of the Henninger medium in root exudates and filtered through 0.2 µm filters. Liquid cultures were then prepared as described for the untreated samples.

### Preparation of *Fagus sylvatica* root exudate

Seeds of European beech (*Fagus sylvatica* L.) were germinated and grown in root trainers with sterilized vermiculite for 2 months at 20°C and light conditions of 250 µmol m^−2^s^−1^ photosynthetic photon flux density (PPFD) (14 hours of day length). Three days before the beginning of the experiment, the seedlings were carefully removed from the containers. The roots were rinsed of the substrate and seedlings were placed in tubes containing 50 mL of deionized water at 20°C for two days at 12 hours photoperiod (light condition: 250 µmol m^−2^s^−1^ PPFD). After 2 days, the plants were discharged and the solution containing the root exudate were filtered (Whatman paper filters, 150 mm) and stored at −20°C until use. Root exudates were assayed for their ability to attract *P. plurivora* zoospores in a capillary assay. To this aim, the zoospore attraction was evaluated by placing a capillary-tube filled with 5 µl of root exudate or water as controls into a zoospore suspension (1×10^5^ spores/mL).

### Sample preparation

Lyophilized *P. plurivora* culture filtrates and *F. sylvatica* root exudate were resuspended in 20 mL of MilliQ water and centrifuged at 5000 *g* for 15 min at 4°C. Samples were desalted and concentrated by using Amicon Ultra centrifugal filters devices with a 3 kDa cut-off (Millipore Corporation, Billerica, MA, USA) according to manufacturer’s instructions. Protein concentration was determined by the bicinchoninic acid (BCA) assay according to manufacturer’s instructions (Thermo Fisher Scientific Pierce, Rockford, IL USA).

### *In-solution* tryptic digestion

Equal aliquots of proteins (50 µg) from *Phytophthora* samples were lyophilized and resuspended in 100 µL of 0.1 M triethylammonium hydrogen carbonate (TEAB) buffer pH 8.0. An equal amount (1 µg) of bovine β-Lactoglobulin (LACβ) was spiked in each sample to serve as an internal standard for experimental bias correction. Proteins were reduced by adding 1 µL of 1% SDS and 2 µL of 50 mM tris (2-carboxyethyl) phosphine (TCEP) and heating at 60°C for 1 h. Free thiol groups of cysteine residues were alkylated by adding 1 µL of 400 mM iodoacetamide and incubating for 30 min at room temperature in the dark with gentle agitation. Proteins were then digested overnight at 37°C with trypsin in 0.1 M TEAB pH 8.0 (protein/trypsin ratio 50∶1 w/w). *F. sylvatica* root exudate were processed as described above.

### iTRAQ labeling and peptide fractionation by OFFGEL electrophoresis

The resulting peptides were tagged with the isobaric tags for relative and absolute quantitation (iTRAQ) reagents Multiplex Kit (AB Sciex, Foster City, CA, USA). Each sample was labeled with one of three isobaric tags reconstituted with 50 µL of isopropanol. The reaction was left to stand at room temperature for 60 min and then blocked by incubating with 8 µL of hydroxylamine 5% for 15 min. The mixtures of labeled peptides were then pooled and dried under vacuum. The lyophilized peptides were dissolved in 800 µL of 5% CH_3_CN/0.1% formic acid (FA), and loaded (2×400 µL) onto C_18_ Macro SpinColumns (Harvard Apparatus). Elution was performed with 2×200 µL of 50% CH_3_CN/0.1% FA. The samples were then dried under vacuum and dissolved in 360 µL of deionized water. A solution containing 6% glycerol and 0.3% IPG buffer pH 3–10 (Agilent, Santa Clara, CA, USA) was added to a final volume of 1.8 mL. Peptides were fractionated according to their p*I* on an Agilent 3100 OFFGEL fractionator using commercial 12 cm IPG pH 3–10 linear strips (GE Healthcare, Waukesha, WI, USA). The strips were rehydrated with 20 µL of rehydration solution (4.8% glycerol, 0.24% IPG buffer pH 3–10) per well. After a 30 min incubation, 150 µL of the sample solution was loaded per well. The isoelectric focalization was carried out at 20°C until a total voltage of 20 kV/h with a maximum current of 50 µA and a maximum power of 200 mW. After the focalization, peptide fractions (12/for each group) were recovered in separate tubes and pH values were measured to check for the efficiency of the pH gradient. Fractions were then dried under vacuum, dissolved in 300 µL of 5% CH_3_CN/0.1% FA, and loaded (2×150 µL) onto C18 Micro SpinColumns (Harvard Apparatus). Elution was performed with 2×100 µL of 50% CH_3_CN/0.1% FA and eluted fractions were dried under vacuum and stored at −20°C until MS analysis.

### Liquid chromatography-tandem mass spectrometry

Lyophilized peptides obtained from OFFGEL fractionation were dissolved in 8 µL of 5% CH_3_CN/0.1% FA; 5 µL of the resulting sample were injected for LC-MS/MS analysis. MS analysis was performed on a LTQ Orbitrap Velos Pro from Thermo Electron (San Jose, CA) equipped with a NanoAcquity UPLC system from Waters (Milford, MA, USA). Peptides were trapped on a home-made (5 µm 200 Å Magic C18 AQ 0.1×2 mm) pre-column (Michrom, Auburn, CA, USA) and separated on a home-made (5 µm 100 Å Magic C18 AQ, 0.75×15 mm) column (Michrom). The analytical separation was run for 65 min using a gradient of 99.9% H_2_O/0.1% FA (solvent A) and 99.9% CH_3_CN/0.1% FA (solvent B). The gradient was run as follows: 0–1 min 95% A and 5% B, then to 65% A and 35% B at 55 min, and 20% A and 80% B at 65 min at a flow rate of 220 nL/min. For MS survey scans, the OT resolution was set to 60000 and the ion population was set to 5×10^5^ with an *m/z* window from 400 to 2000. A maximum of 3 precursors was selected for both the collision-induced dissociation (CID) in LTQ and the high-energy C-trap dissociation (HCD) with analysis in the OT. For MS/MS in the LTQ, the ion population was set to 7×10^3^ (isolation width of 2 *m/z*) while for MS/MS detection in the OT, it was set to 2×10^5^ (isolation width of 2.5 *m/z*), with resolution of 7500, first mass at *m/z* = 100, and maximum injection time of 750 ms. The normalized collision energies were set to 35% for CID and 60% for HCD.

Data extraction, database interrogation and relative protein quantification. Peak lists were generated from raw data using the embedded software from the instrument vendor (extract_MSN.exe v5.0). After peaklist generation, the CID and HCD spectra were merged for simultaneous identification and quantification by using EasyProtConv [24]. The merged mgf files, combined from the 12 analyzed OFFGEL fractions, were used for protein identification and quantification with EasyProt software platform v2.2. [24] For protein identification, parameters were specified as follows: databases = uniprot_sprot (2013_12 of 11-Dec-2013)/uniprot_trembl (2013_12 of 11-Dec-2013); taxonomy = *Phytophthora*; precursor error tolerance = 25 ppm; variable modification = oxidized methionine; fixed modifications = carbamidomethylated cysteine, iTRAQ-labeled amino terminus and lysine; enzyme = trypsin; potential missed cleavage = 2; cleavage mode = normal; search round = 1, scoring model = CID_LTQ_scan_LTQ; instrument type = ESI-LTQ-Orbitrap. Protein and peptide scores were set up to maintain the false positive peptide ratio below 5%. For protein quantification, the isotopic correction was applied to reporter intensities according to the iTRAQ reagents certificate of analysis. iTRAQ reporter peak intensities were further normalized using the spiked LACβ standard. For each protein, the mean, the standard deviation, and the coefficient of variation (CV) of relative peptide intensities were obtained for the two experimental groups by using the EasyProt Mascat quantification module that computes a per-peptide ratio from the reporter ion abundance values for the given peptide [24]. The ratio of a protein is then computed as the geometric mean of all peptide ratios belonging to the protein. A Student's t-test distribution, with a null hypothesis stating that the log2 of the protein ratio is equal to zero (confidence interval = 95%) was computed by the algorithm.

### Bioinformatics analyses

Proteins with a predicted N-terminal signal sequence were identified by using the SignalP 4.1 server [25] available at http://www.cbs.dtu.dk/services/SignalP/. The similarity search for uncharacterized proteins deriving from ORFs was performed by using the Blast tool at http://www.uniprot.org/blast/. Sequences were aligned using Clustal W2 [26], rendered with Jalview [27] and manually annotated as previously reported [28]. Protein domains in selected effectors were identified using Interpro tool [29].

### Nuclear Magnetic Resonance (NMR) analyses

*F. sylvatica* root exudate (40 mg) was transferred to a 2 mL microtube and analysed. Samples for NMR analysis were prepared in a mixture of 90 mM phosphate buffer pH 6.0 (Fluka Chemika, Buchs, Switzerland) in D_2_O (Cambridge Isotope Laboratories, Tewksbury, MA, USA) containing 0.01% w/w trimethylsilylpropionic-2,2,3,3-*d_4_* acid sodium salt (TMSP, Sigma-Aldrich) and methanol-*d_4_* (Sigma-Aldrich). A volume of 1.5 mL of phosphate buffer in D_2_O and methanol-*d_4_* (1∶1) was added to the samples. The mixtures were vortexed at room temperature for 1 min, ultrasonicated (Elma Transonic Digitals, Singen, Germany) for 40 min and centrifuged at 15800 *g* for 10 min. Aliquots of samples (0.6 mL) were transferred to an NMR tube and analysed.

Organic components from *P. plurivora* culture filtrates were partially purified on amberlite XAD4 washed with water and eluted with methanol. The MeOH eluate was dried, dissolved in phosphate buffer in D_2_O and methanol-*d_4_* (1∶1), and analysed by NMR.

NMR spectra were recorded at 25°C on a 300.03 and 500 MHz for ^1^H on a Varian Mercury Plus 300 Fourier transform NMR. CD_3_OD was used as the internal lock. Each ^1^H NMR spectrum consisted of 256 scans with the following parameters: 0.16 Hz/point, acquisition time (AQ) = 1.0 s, relaxation delay (RD) = 1.5 s, 90° pulse width (PW) = 13.8 µs. A presaturation sequence was used to suppress the residual H_2_O signal. FIDs were Fourier transformed with LB = 0.3 Hz. The resulting spectra were manually phased, baseline-corrected and calibrated to TMSP at 0.0 ppm. ^1^H-^1^H correlated spectroscopy (COSY), heteronuclear single quantum coherence (HSQC) and heteronuclear multiple bond correlation (HMBC) spectra were recorded. COSY spectra were acquired with a 1.0 s relaxation delay and 2514 Hz spectral width in both dimensions. The window function for COSY spectra was sine-bell (SSB = 0). HSQC and HMBC spectra were obtained with a 1.0 s relaxation delay and 3140 Hz spectral width in f2 and 18116 Hz in f1. Qsine (SSB = 2.0) was used for the window function of the HMBC. The optimized coupling constants were 140 Hz for HSQC and 8 Hz for HMBC. The main organic acids of *F. sylvatica* root exudate were identified based on the comparison with spectra collected from pure standards and further confirmed by spiking the sample with standard compounds.

### Amino acid analysis of *F. sylvatica* root exudate

For the analysis of free amino acids, aliquots of lyophilized *F. sylvatica* root exudate (20 mg) were precipitated with 80% cold ethanol (1 mL) in the presence of nor-Leu (50 nmol) as internal standard. The sample was homogenized with a teflon pestle and centrifuged at 15800 *g* for 30 min at 4°C. The supernatant was lyophilized and then treated with 3% sulfosalicylic acid (500 µL). Following centrifugation at 15800 *g* for 30 min at 4°C, the supernatant was directly analyzed on a Biochrom 20 amino acid analyser (Biochrom, Cambridge, U.K.), equipped with a post-column ninhydrin derivatization system. Aliquots of samples (25 µL) were analyzed in duplicate as previously reported [30].

## Results

### Characterization of *P. plurivora* secretome by proteomic analysis

To perform a comprehensive profiling of *P. plurivora* secretome and to investigate the occurrence of quantitative changes in protein levels following interaction with the root exudate of *Fagus sylvatica*, a strategy based on isobaric tags for relative and absolute quantitation (iTRAQ) was exploited. By high-resolution LC-MS/MS, 448 unique peptides were assigned to 272 proteins by the EasyProt algorithm using the *Phytophthora* species-specific uniprot/trembl database (Table S1). According to a widely adopted approach in proteomic research, the high-throughput identification of gene products from non-model organisms such as *Phytophthora* species was performed by homology database search for orthologous proteins [31]. The entire data set of proteins was filtered by considering only identifications with a minimum of two peptides, yielding a list of 103 proteins (Table S2). However, single peptide-based identifications were also considered if proteins matched with known *Phytophthora* effectors (Table S2).

**Figure 1:**
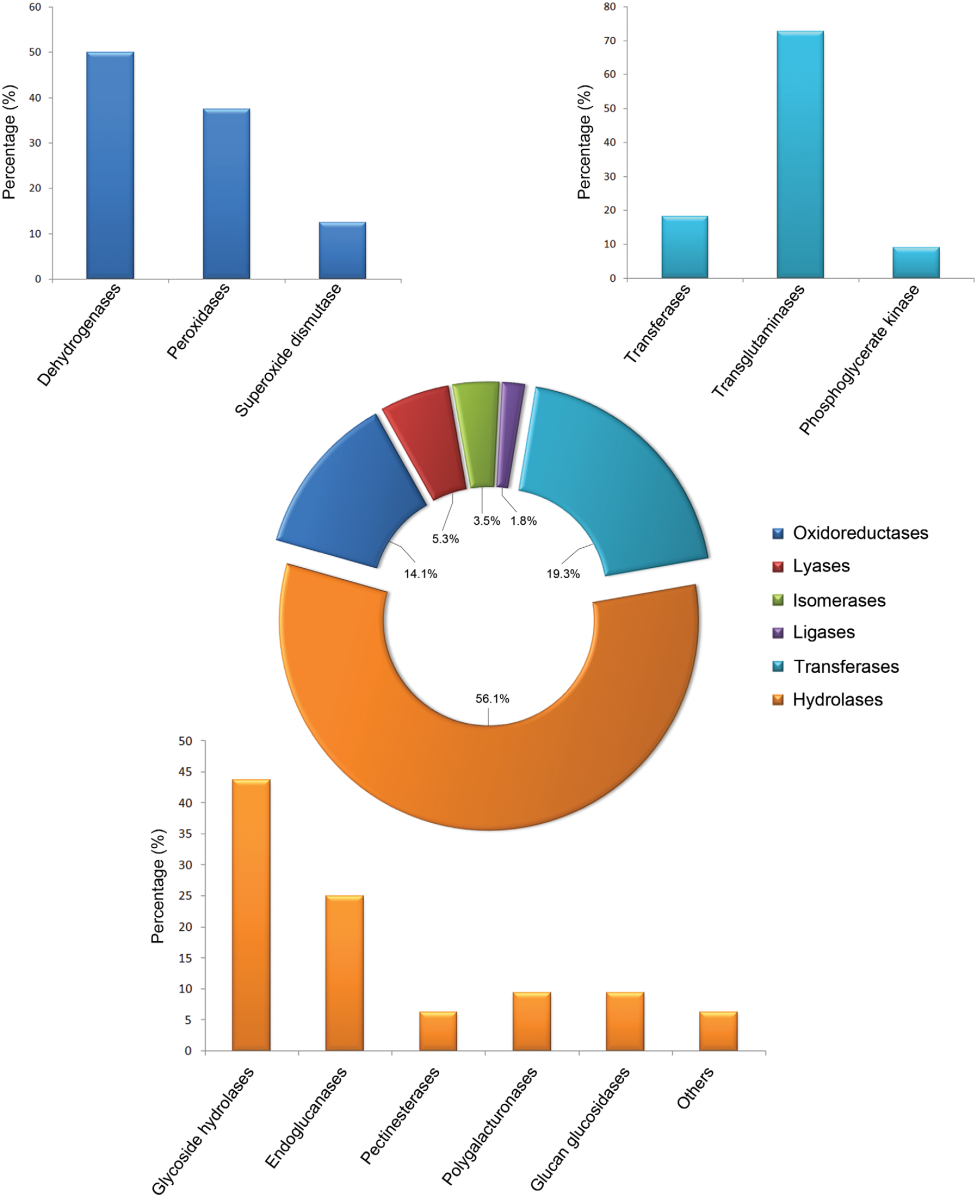
Schematic representation of proteins with enzymatic activity enriched in *P. plurivora* secretome. The most represented categories for the oxidoreductases, transferases and hydrolases are also reported.

The proteins identified in culture filtrates of *P. plurivora* by LC MS/MS were analyzed for classical (i.e. signal peptide-driven secretion through the ER/Golgi pathway) secretion pathways, revealing that about 60% were endowed with the N-terminal signal sequence for extracellular secretion. Furthermore, in order to associate a putative function to uncharacterized proteins deriving from ORFs, a similarity search was performed by using the BLASTP software. By this approach, several proteins were matched to accession numbers of annotated proteins with a percentage of identity of amino acid sequences above 60% (Table S2). A high percentage of proteins identified in the *P. plurivora* secretome was previously predicted to be secreted by using genomic and/or bioinformatic strategies (Table 1). Our results thus provide a direct experimental confirmation of the presence of some putative effectors within the *P. plurivora* culture filtrates. We also found that proteins with enzymatic activity (55.3%) were highly enriched in *P. plurivora* secretome, with oxidoreductases (14.1%), transferases (19.3%) and hydrolases (56.1%) being the most represented categories (Figure 1). Among hydrolases, several cell-wall-degrading enzymes (e.g. glycoside hydrolases, endoglucanase, pectinesterases, polygalacturonases and glucan hydrolases) were identified (Figure 1). After that, we investigated the occurrence of changes in the amount of *P. plurivora* proteins following treatment with the root exudate of *Fagus sylvatica*. The isobaric tag–based quantification allowed the detection of 21 proteins with differential amounts in *P. plurivora* culture filtrate following treatment with root exudate compared to the untreated *P. plurivora* sample (Table S3). In particular, among the proteins down-regulated in *P. plurivora* culture filtrate treated with the *F. sylvatica* root exudate, the highest differences occurred for the putative D-isomer specific 2-hydroxyacid dehydrogenase, several known *Phytophthora* effectors (e.g. NLP effector, Avr1b-1 avirulence-like proteins and transglutaminase elicitors) and proteins with glycoside hydrolase, pectate lyase and glucanase activities.

**Table 1:** List of effectors identified in the *P. plurivora* secretome by high resolution LC MS/MS.

Uniprot AC	Blast Match AC (% identity)	Species	Protein description	SignalP	References
G4ZKR2		*P. sojae*	Avr1b-1 avirulence-like protein	Y	[11]
H3GRA9	G4ZKR2 (77)	*P. sojae*	Avr1b-1 avirulence-like protein	Y	[11]
H3GRB0	G4ZKR2 (70)	*P. sojae*	Avr1b-1 avirulence-like protein	Y	[11]
G4ZK12	G4ZKR2 (69)	*P. sojae*	Avr1b-1 avirulence-like protein	Y	[11]
D0MXJ2		*P. infestans*	Berberine-like protein	Y	[28]
D0N574		*P. infestans*	Berberine-like protein	Y	[28]
G4YQ65	D0MXJ2 (86)	*P. infestans*	Berberine-like protein	Y	[28]
G4Z7E3	D0N574 (72)	*P. infestans*	Berberine-like protein	Y	[28]
P15569		*P. cinnamomi*	Beta-elicitin cinnamomin	N	[72]
G4ZY09	D0NWB4 (87)	*P. infestans*	Carbonic anhydrase	N	[28]
H3GIU0	D0NW76 (76)	*P. infestans*	Carbonic anhydrase	Y	[28]
O42830		*P. parasitica*	CBEL protein, formerly GP34	Y	[11]
D0MY47		*P. infestans*	Cellulose binding elicitor lectin (CBEL)	N	[11]
Q9AT01		*P. capsici*	Elicitin	N	-**
Q3L578		*P. megakarya*	Necrosis and ethylene-inducing protein 1	N	[11]
Q3L570		*P. megakarya*	Necrosis and ethylene-inducing protein 7	N	[11]
G4ZP65	G4ZA69 (67)	*P. sojae*	Necrosis inducing-like protein NPP1 type	Y	[11]
Q8LKL0		*P. sojae*	Necrosis-inducing-like protein	Y	[11]
T2FFK2		*P. capsici*	NLP effector	Y	[11]
G2XKV6		*P. capsici*	Pectate lyase	Y	[28]
T1NXE7		*P. capsici*	Pectate lyase	Y	[28]
G2XKU9		*P. capsici*	Pectinesterase	Y	[28]
G2XKV0		*P. capsici*	Pectinesterase	Y	[28]
G2XKV3		*P. capsici*	Pectinesterase	Y	[28]
H3GDN4	D0NSG4 (65)	*P. infestans*	SCP-like extracellular protein	Y	[73]
G4ZZW1	B0B0Q5 (81)	*P. cinnamomi*	Transglutaminase elicitor	Y	[52]
G5A054	Q6XDM3 (74)	*P. infestans*	Transglutaminase elicitor M81C	N	[52]
H3G7W2	Q6XDM3 (76)	*P. infestans*	Transglutaminase elicitor M81C	Y	[52]
Q6XDM3		*P. infestans*	Transglutaminase elicitor M81C	Y	[52]
G4ZZV6	D0NUH0 (64)	*P. infestans*	Transglutaminase elicitor	N	[52]
G4ZZW4	D0NUH0 (64)	*P. infestans*	Transglutaminase elicitor	N	[52]
H3GZF4*	D0NUH0 (70)	*P. infestans*	Transglutaminase elicitor	Y	[52]
H3GZF6*	D0NUH0 (60)	*P. infestans*	Transglutaminase elicitor	Y	[52]
D0NAC8	D0NUH1 (84)	*P. infestans*	Transglutaminase elicitor-like protein	N	[52]
D0RLV7		*P. infestans*	Transglutaminase elicitor-like protein	N	[52]

*Sequences containing the RXLR motif.

**Nucleotide sequence submitted to the EMBL/GenBank/DDBJ databases.

The secretion prediction according to signal peptide probability of Signal P 4.1 server is reported. Y and N indicate the presence or absence of the signal peptide for secretion, respectively.

### Characterization of *Fagus sylvatica* root exudate by NMR and amino acid analysis

**Figure 2:**
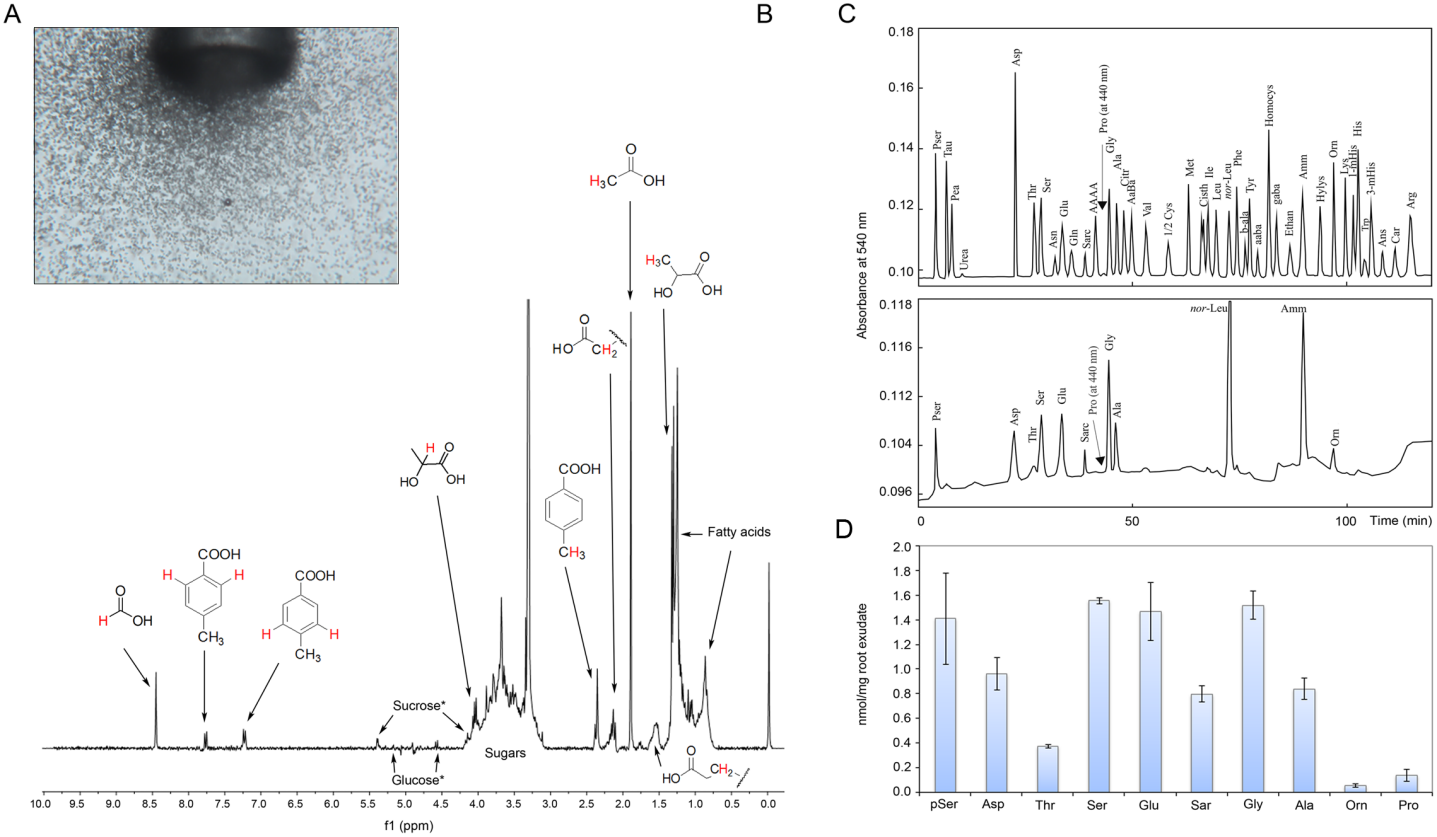
*Fagus sylvatica* root exudate characterization. A) Representative photograph shoving the ability of *Fagus sylvatica* root exudate to attract *P. plurivora* zoospores. B) ^1^H NMR spectrum of *F. sylvatica* root exudate acquired at 300.03 MHz in methanol-d4-buffer phosphate 1∶1. Protons responsible for NMR signals of molecules are highlighted in red in the structures. Signals of anomeric protons are marked with asterisks. C) Free amino acid profile of *F. sylvatica* root exudate (lower panel) compared to standards (upper panel). D) Bar chart showing the amount (nmol/mg of root exudate) of free amino acids detected in the *F. sylvatica* root exudate.

It is now widely recognised that, beside proteins, both plant roots and pathogens secrete small molecular weight compounds mediating biological interactions occurring in the rhizosphere. Given the ability of *Fagus sylvatica* root exudate to attract *P. plurivora* zoospores (Figure 2A), we characterized its main low-molecular-weight components by NMR analysis. The ^1^H-NMR spectrum of *Fagus sylvatica* root exudate showed signals belonging to several organic acids (Figure 2B) including formic acid (singlet at δ 8.47) and acetic acid (singlet at δ 1.91). Furthermore, signals belonging to lactic acid were detected as a doublet at δ 1.34 (H3, J = 6.9 Hz) and a quartet at δ 4.06 (H2, J = 6.9 Hz) in 3∶1 ratio. Signals characteristic of fatty acids were also detected (triplet at δ 0.88, correlating in a COSY experiment with a signal at δ 1.29). In this instance, further correlations attributable to the carboxylic end of fatty acids (C2 and C3, respectively) were revealed among a signal at δ 2.16 (triplet J = 7.5 Hz) and a multiplet at δ 1.55 (both methylenic). Typical signals diagnostic for the presence of sugars (glucose and sucrose) were also detected. Furthermore, two signals in the aromatic region at δ 7.23 and 7.79 (J = 7.8 Hz), along with a singlet methyl at δ 2.37 allowed identifying *p-*toluic acid within the *Fagus sylvatica* root exudate. This hypothesis was confirmed by the COSY correlations among the aromatic protons, and among that at δ 7.23 with the methyl protons.

Since amino acids represent an important class of the organic low-molecular-weight compounds present in plants root exudates, we also determined the free amino acids composition of the *Fagus sylvatica* root exudate. Among the 48 amino acids and derivatives detectable with the used methodology, 10 amino acids were revealed within the exudate (Figure 2C and 2D). Of these, the most abundant were pSer, Asp, Ser, Glu, Sar, Gly and Ala (range 0.80–1.56 nmol/mg of root exudate). We also detected lower amounts of Orn (0.06 nmol/mg), Pro (0.14 nmol/mg) and Thr (0.38 nmol/mg).

### Characterization of *P. plurivora* culture filtrates by NMR

**Figure 3:**
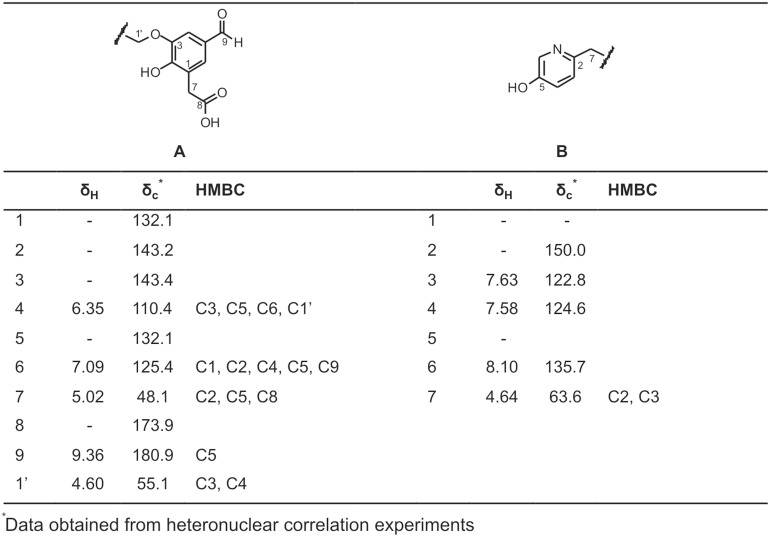
^1^H, ^13^C NMR data and HMBC correlations of metabolites A and B.

A characterization of low-molecular-weight compounds by NMR was also performed on *P. plurivora* culture filtrates. Low abundant organic metabolites, not previously structurally characterized in *Phytophthora* were identified by ^1^H-NMR and 2D-NMR. An aromatic moiety was observed on the basis of two meta-coupled protons (J = 2.5 Hz), at δ 6.35 and at δ 7.09 for the most abundant metabolite of class A. The correlations detected in an HMBC experiment (Figure 3) allowed to determine the functional groups bound to this aromatic skeleton, as well as their position: a formyl (δ_H_ 9.36, s) and an acetyl (δ_H_ 5.02, s) moieties and two hydroxyl groups. Furthermore, correlations among the methylene protons at δ 4.60 (singlet) with the C3 and C4 carbons were diagnostic of its linkage with the oxygen at C3 position. The presence of similar signals in the aromatic region allowed the detection of further structural analogues: a second system constituted by two meta-coupled protons at δ 6.39/7.15 and an aldehydic signal at δ 9.31 and two further systems at δ 6.39/7.21/9.19 and 6.03/6.65/9.40. However, due to their low abundance in the extract, it was not possible to determine the substituents of the aromatic ring. Moreover, characteristic signals in the aromatic region were in agreement with a substituted pyridine structure of metabolite B (Figure 3).

Beyond these aromatic compounds, signals belonging to organic acids, likely esterified to other units were also detected. These moieties were identified as succinic acid (a singlet proton at δ 2.61), 3-hydroxyisovaleric acid (a singlet at δ 1.30 and a singlet methylene at δ 2.49) and isovaleric acid (a methyl at δ 0.98, a methine at δ 1.76 and a methylene at δ 1,75).

## Discussion

The coevolutionary dynamics between pathogens and host plants result in the development of reciprocal adaptation strategies involved in the interaction [19]. Oomycetes, including *Phytophthora* species, have evolved advanced pathogenicity mechanisms mainly based on the secretion of effectors that target host plant apoplast and cytoplasm [9]–[13]. Functionally, these molecules suppress plant defenses and promote host colonization.

A recent breakthrough in effector biology gain insight into the molecular basis of adaptation and specialization of pathogen effectors following host colonization. It has been demonstrated that polymorphisms within the amino acid sequences of orthologous protease inhibitors from *Phytophthora infestans* and *Phytophthora mirabilis* are key elements in determining the specificity to protease targets of their respective host plants [32]. These findings highlight the need to further expand our knowledge on structural and functional properties of effectors. Over the past decade, big strides have been made toward the categorization of these effectors and putative proteins secreted by *Phytophthora* species [9]–[13]. Genetic and bioinformatic approaches, often complemented by biochemical and functional studies, allowed obtaining lists of effectors potentially involved in host-pathogen interaction [20], [21], [28], [33]. Several candidate genes identified by data mining tools on the basis of specific criteria (e.g. presence of N-terminal signal sequence and/or conserved consensus motifs for cellular sorting) have subsequently been validated by functional assays such as expression in plants and evaluation of effector-like activities [10]–[13], [34], [35]. However, since functional studies require many efforts to be performed in a high-throughput manner and signal peptides are not always present in secreted proteins, many unpredictable effectors and secreted proteins could remain largely underestimated.

Here we provide a direct evidence of the secretion of a large number of proteins by *P. plurivora*. We exploited an analytical strategy that relies on the high accuracy of LTQ-Orbitrap LC-MS/MS systems for peptide *de novo* sequencing and high-throughput protein identification by database search. This approach, previously applied by us to the secretome characterization of several cell lines and human primary cultures [36]–[40], proved to be suitable for identifying proteins in culture filtrate of *P. plurivora*.

We identified a subset of proteins characterizing the *P. plurivora* secretome, several of which were found to be cell-wall-degrading enzymes. Consistent with our findings, previous studies reported that, at the infection sites, a combination of mechanical pressure and release of cell-wall degrading enzymes allows the plant wall being breached by a penetration peg which develops into hyphae that ramify through the plant tissue [41]–[43]. Accordingly, cell-wall degrading enzymes have been previously identified in culture media of filamentous pathogens [44], [45]. By expressed sequence tag (EST) analysis, it has been shown that zoospores already contain transcripts for several secreted plant-cell wall-degrading enzymes including cutinases, polygalacturonases, pectate lyases and cellulases (β-1,4-glucanases) [41]. Furthermore, several glucanases are specifically expressed in germlings and hyphae [46].

**Figure 4:**
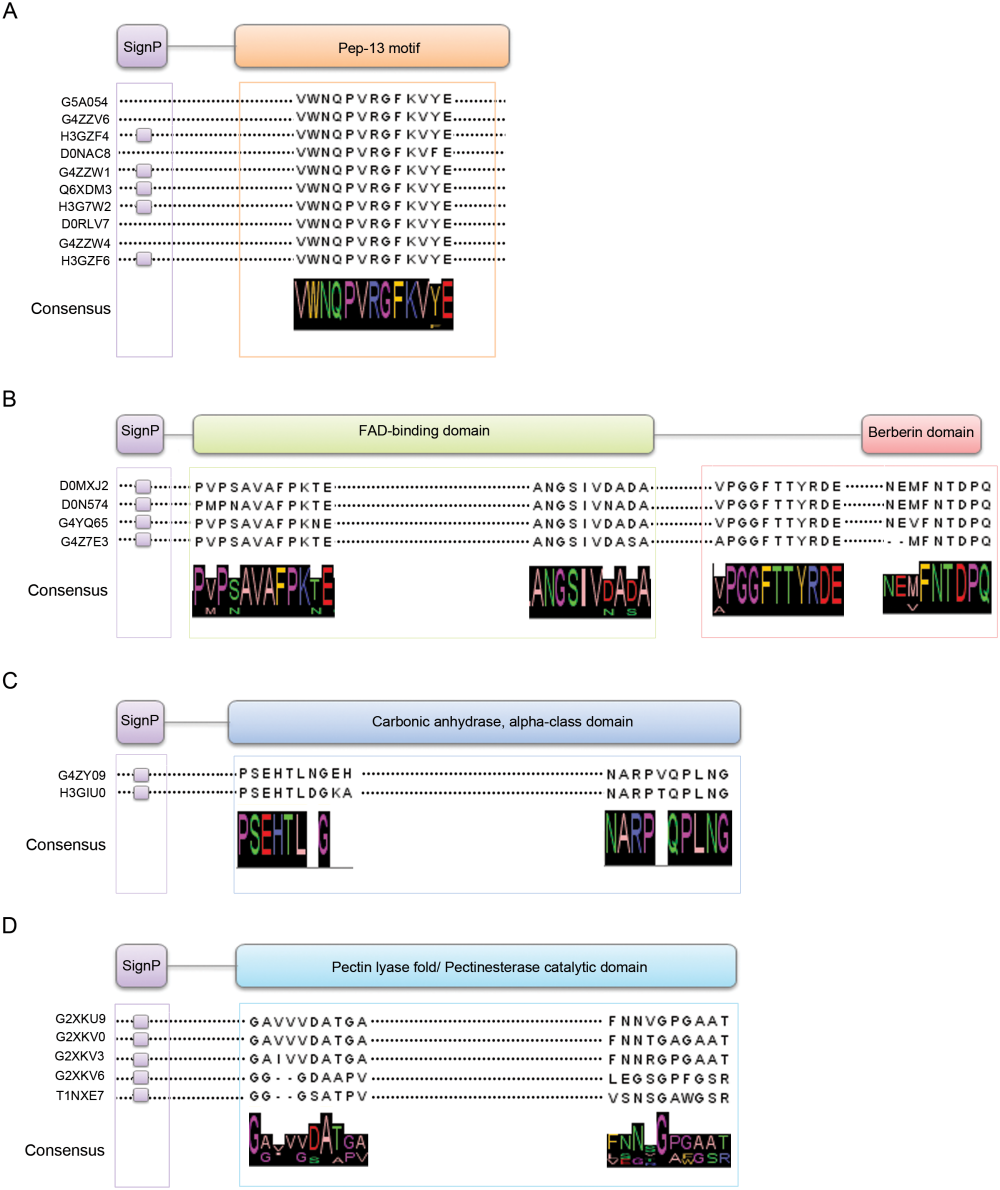
Multiple sequence alignments showing similarity and conserved domains identified by the Interpro resource available at http://www.ebi.ac.uk/interpro/in A) Pep-13 transglutaminase peptide elicitors (according to Brunner et al., 2002 [52]), B) berberin-like proteins (Pfam PF08031), C) carbonic anhydrases (PROSITE PS51144; Pfam PF00194) and D) pectinesterase/pectate lyases (Pfam PF01095/SUPERFAMILY SSF51126). Signal sequences at N-terminus (SignP) and the consensus sequences are also reported.

In addition, several effectors within the *P. plurivora* secretome were also identified by performing a *Phytophthora* species-specific database search or by a BLASTP similarity search for uncharacterized proteins deriving from ORFs. Among them, apoplastic effectors belonging to the necrosis and ethylene-inducing protein 1 (NEP1) family were detected. NEP1-like proteins (NLPs) are highly conserved proteins of about 25 kDa widely distributed in bacteria, fungi and oomycetes [47]–[49]. Several members of this family are able to induce cell death in many dicotyledons [47]. Although their contribution in pathogenicity is still unclear, an important role in necrosis-inducing activity has been postulated based on NLPs phylogenetic conservation and broad-spectrum activity. An involvement of NLPs effectors in facilitating host colonization has been hypothesized due to their late expression during the necrotrophic phase of host infection in *P. sojae* and *P. infestans* [48]. Several transglutaminase elicitor isoforms were also identified, some of which carrying the RXLR motif, known to be involved in delivering the effector protein into the host cell [14], [50], [51]. An alignment of the identified transglutaminase (TGases, Figure 4A) showed the presence of the invariant Pep-13 motif, reported to be highly conserved in several *Phytophthora* species [52]. Previous studies reported that GP42, an abundant cell wall glycoprotein of *P. sojae* able to trigger plant defense, was a Ca^2+^-dependent TGase. It is currently unknown if TGases play a key role in *Phytophthora* virulence [11]. Other apoplastic effectors belonging to elicitin protein family and CBEL elicitors (Cellulose Binding Elicitor and Lectin-like) were identified. Elicitins are well-characterized small secreted proteins inducing defense responses in specific plants (i.e. hypersensitive cell death and resistance against subsequent pathogen attack) for which a role as extracellular sterol carriers has been reported [53]–[59]. Similarly, the ability to induce defense gene expression in tobacco plants was also reported for CBEL elicitor, a 34-kDa cell wall protein initially isolated from *Phytophthora parasitica* var. *nicotianae* [60].

Among cytoplasmic effectors, we identified several isoforms of avirulence-like proteins (Avr), that are well-studied effectors able to activate host defense responses and innate immunity [11], [34]. However, an essential role for virulence was demonstrated for the *P. infestans* RXLR effector Avr3a which is able to suppress hypersensitive cell death induced by INF1 elicitin [34]. This dual mode of action suggests that Avr proteins, as well as probably other *Phytophthora* effectors, are only beginning to be understood. Further functional studies are needed to unravel the molecular mechanisms through which oomycetes effectors manipulate and reprogram plant defense for host cells colonization.

Interestingly, we also provide the experimental evidence of the presence within *P. plurivora* secretome of some putative effectors so far predicted by *in silico* approaches [28]. Among these novel candidate virulence factors in *P. infestans*, we identified highly conserved isoforms of the pectinesterase/pectate lyase, carbonic anhydrase and berberin-like protein family (Figure 4B–D). The pectinesterase/pectate lyase enzymes participate in the degradation of the pectic components of plant cell walls [28], [61].

Berberine-bridge enzymes (BBEs) are flavoenzymes found in archaea, bacteria, plants and fungi that catalyze carbohydrate oxidation in plants [28]. They are involved in the generation of reactive oxygen species, including H_2_O_2_, and in the synthesis of alkaloids in plants. In addition, it has been predicted that the *P. infestans* genome encodes 13 carbonic anhydrases (α-CAs), seven of which are putatively secreted [28]. Both BBEs and α-CAs oxidoreductases are likely involved in pathogen detoxification processes and triggering host cell death responses [28]. In a recent study, Meijer and coworkers confirmed the secretion of five berberine-like proteins in *P. infestans* by using a proteomic approach [62]. Although their function in *Phytophthora* has not been fully elucidated, it has been hypothesized that they may act as virulence factors or as protecting agents against plant counter defenses [28], [62], [63].

For a subset of the identified known effectors (e.g. NLP effector, Avr1b-1 avirulence-like proteins and transglutaminase elicitors) as well as for selected proteins with glycoside hydrolase, pectate lyase and glucanase enzymatic activity, a lower amount was observed in *P. plurivora* culture filtrate following treatment with the *F. sylvatica* root exudate compared to the untreated sample, thus providing a list of candidate secreted proteins whose expression and/or secretion is affected following interaction with components of the host root exudate.

This finding prompted us to integrate the proteomic profiling of the *P. plurivora* secretome with a low-molecular-weight profile by NMR and amino acid analysis of the *F. sylvatica* root exudate with the primary aim to collect information on its composition. Chemical signaling between plant roots and phytopathogens is often based on root-derived chemicals [64], [65]. Among them, the most abundant components include low-molecular weight compounds such as amino acids, organic acids, sugars, phenolics, and other secondary metabolites [64], [65]. Accordingly, among the main components of the *F. sylvatica* root exudate, we found some organic acids such as formic acid, acetic acid, lactic acid and *p-*toluic acid. In addition, we identified pSer, Asp, Ser, Glu, Sar, Gly and Ala as the most abundant amino acids within the root exudate. Investigations with *Phytophthora* and *Pythium* species demonstrated that zoospores are attracted by chemicals present in root exudates, mainly amino acids and sugars [66]–[68]. The stronger response of zoospores attraction was found for dicarboxylic amino acids (i.e. glutamic acid, aspartic acid and 4-aminobutyric acid), although the attraction toward a wide range of compounds including sugars and organic acids was also observed [68], [69]. In addition, it has been reported that specific isoflavones released by soybean roots into the rhizosphere were chemoattractants for *P. sojae* zoospores [5], [70], [71]. Furthermore, since no NMR data are available on low-molecular-weight compounds released by *Phytophthora* species, we performed a direct analysis of organic components also on *P. plurivora* culture filtrates. Although their low abundance did not allow a comprehensive structural elucidation, not previously described aromatic structures were identified by ^1^H-NMR and 2D-NMR analyses. We also identified succinic acid, 3-hydroxyisovaleric acid and isovaleric acid, likely esterified to other components of the extract.

Overall, the reciprocal influence of molecules secreted by *Phytophthora* and plants in host-pathogen interaction is extremely complex and far from being fully understood. It is now evident that an array of extracellular signals contributes to oomycete pathogenicity and that synergistic effects of effectors and chemicals may play an important role in the phenomenon.

Here we provide a picture of the *P. plurivora* secretome, by simultaneously identifying the highest number of proteins so far reported by direct biochemistry approaches. For some of them a different amount was detected following interaction with the host root exudate. The knowledge of mechanisms regulating these responses, however, is still incomplete also due to the difficulty of studying functional effects of a large number of effectors. Nevertheless, the shot-gun LC-MS/MS methodology proved to be successful for obtaining a comprehensive profiling of *P. plurivora* secretome. We believe that the application of this strategy, complementary to genomic and bioinformatic approaches, to secretome characterization of other *Phytophthora* species will enable to deepen our understanding of different virulence strategies of pathogens and unravel their role in plant immunity.

## Supporting Information

Table S1 **Details of high resolution LC MS/MS data.** By high-resolution LC-MS/MS, 448 unique peptides were assigned to 272 proteins by the EasyProt algorithm using the Phytophthora species-specific uniprot/trembl database.
(XLS)

Table S2 **Proteins identified in the** ***P. plurivora*** **secretome by high resolution LC MS/MS.** The higher number of unique peptides for each protein identification is reported. Single-peptide identifications have been only considered when proteins matched with known Phytophthora effectors. The secretion prediction according to signal peptide probability of Signal P 4.1 server is reported; Y and N indicate the presence or absence of the signal peptide for secretion.
(DOCX)

Table S3 **List of proteins detected with differential amount in** ***P. plurivora*** **culture filtrates (He) following treatment with the root exudate of** ***Fagus sylvatica*** **(He+RE).** The quantification was performed by isobaric labeling coupled to LC-MS/MS analysis^a^. The secretion prediction according to signal peptide probability of Signal P 4.1 server is reported; Y and N indicate the presence or absence of the signal peptide for secretion.
(DOCX)
