# Supplementary material for: Molecular Profiling of the Phytophthora plurivora Secretome: A Step towards Understanding the Cross-Talk between Plant Pathogenic Oomycetes and Their Hosts
Source: PLoS One. 2014 Nov 5;9(11):e112317. doi: 10.1371/journal.pone.0112317 (PMC4221288; doi:10.1371/journal.pone.0112317)
Supplement: Table S2 — Proteins identified in the P. plurivora secretome by high resolution LC MS/MS. The higher number of unique peptides for each protein identification is reported. Single-peptide identifications have been only considered when proteins matched with known Phytophthora effectors. The secretion prediction according to signal peptide probability of Signal P 4.1 server is reported; Y and N indicate the presence or absence of the signal peptide for secretion. (DOCX) [file pone.0112317.s002.docx]

**Table S2.** Proteins identified in the *P. plurivora* secretome by high resolution LC MS/MS. The higher number of unique peptides for each protein identification is reported. Single-peptide identifications have been only considered when proteins matched with known Phytophthora effectors. The secretion prediction according to signal peptide probability of Signal P 4.1 server is reported; Y and N indicate the presence or absence of the signal peptide for secretion.

| **Uniprot AC** | **Blast Match AC (% identity)** | **Species** | **Protein description** | **# peptides** | **SignalP** |
| --- | --- | --- | --- | --- | --- |
| G5AHN2 | D0NNQ8 (88) | *P. infestans* | 1,3-beta-glucanosyltransferase | 2 | N |
| D0MSD8 |  | *P. infestans* | 5-methlytetrahydropteroyltriglutamate-homocysteine methyltransferase | 2 | N |
| Q8SAQ8 |  | *P. infestans* | 6-phosphogluconate dehydrogenase | 2 | N |
| Q84P47 |  | *P. megakarya* | Actin A | 2 | N |
| D0P3F1 |  | *P. infestans* | Aldose 1-epimerase | 2 | Y |
| D0N577 |  | *P. infestans* | ATP synthase subunit beta | 4 | N |
| H3GRB0 | G4ZKR2 (70) | *P. sojae* | Avr1b-1 avirulence-like protein | 2 | Y |
| G4ZKR2 |  | *P. sojae* | Avr1b-1 avirulence-like protein | 1 | Y |
| H3GRA9 | G4ZKR2 (77) | *P. sojae* | Avr1b-1 avirulence-like protein | 1 | Y |
| G4ZK12 | G4ZKR2 (69) | *P. sojae* | Avr1b-1 avirulence-like protein | 6 | Y |
| D0MXJ2 |  | *P. infestans* | Berberine-like protein | 3 | Y |
| D0N574 |  | *P. infestans* | Berberine-like protein | 3 | Y |
| G4Z7E3 | D0N574 (72) | *P. infestans* | Berberine-like protein | 4 | Y |
| G4YQ65 | D0MXJ2 (86) | *P. infestans* | Berberine-like protein | 2 | Y |
| P15569 |  | *P. cinnamomi* | Beta-elicitin cinnamomin | 1 | N |
| D0MQS7 |  | *P. infestans* | Calcineurin-like phosphoesterase | 4 | N |
| G4ZY09 | D0NWB4 (87) | *P. infestans* | Carbonic anhydrase | 1 | N |
| H3GIU0 | D0NW76 (76) | *P. infestans* | Carbonic anhydrase | 1 | Y |
| D0N7D7 |  | *P. infestans* | Catalase-peroxidase | 5 | Y |
| H3GL28 | D0N7D7 (100) | *P. infestans* | Catalase-peroxidase | 4 | Y |
| O42830 |  | *P. parasitica* | CBEL protein, formerly GP34 | 2 | Y |
| Q2V8M9 |  | *P. sojae* | Cell 12A endoglucanase | 2 | Y |
| H3G7P9 | Q30BY5 (100) | *P. ramorum* | Cell 12A endoglucanase | 2 | N |
| Q0PQ32 |  | *P. infestans* | Cell 5A endo-1,4-betaglucanase | 2 | N |
| H3H0Z9 | A1YT27 (75) | *P. ramorum* | Cell 5A endo-1,4-betaglucanase | 2 | N |
| G4YRH9 |  | *P. sojae* | Cellulase-1, endo-1,4-beta-glucanase | 4 | N |
| G5A663 |  | *P. sojae* | Cellulase-9, endo-1,4-beta-glucanase | 3 | Y |
| D0MY47 |  | *P. infestans* | Cellulose binding elicitor lectin (CBEL) | 2 | N |
| G4ZCB7 |  | *P. sojae* | Chloroperoxidase | 2 | Y |
| G4ZEQ8 | D0MT61 (92) | *P. infestans* | D-isomer specific 2-hydroxyacid dehydrogenase | 2 | N |
| Q9AT01 |  | *P. capsici* | Elicitin | 1 | N |
| G5AFQ9 | D0N444 (91) | *P. infestans* | Elicitor-like transglutaminase M81-like protein | 1 | N |
| D0MXT1 |  | *P. infestans* | Endo-1,3(4)-beta-glucanase 1 | 7 | Y |
| A7XL58 |  | *P. megakarya* | Enolase | 4 | N |
| A7XLC9 |  | *P. megakarya* | Enolase | 5 | N |
| G4Z0C3 |  | *P. sojae* | Family 3 glycoside hydrolase | 2 | Y |
| B0B0H6 |  | *P. cinnamomi* | Glucan 1,3-beta-D-glucosidase | 2 | Y |
| D0MQ88 |  | *P. infestans* | Glucan 1,3-beta-glucosidase | 5 | Y |
| H3GZF0 | D0NUH5 (79) | *P. infestans* | Glucan 1,3-beta-glucosidase | 3 | Y |
| H3G751 |  | *P. ramorum* | Glucose-6-phosphate 1-dehydrogenase | 2 | N |
| D0NNT6 |  | *P. infestans* | Glutamine synthetase | 2 | N |
| D0MV27 |  | *P. infestans* | Glycoside hydrolase | 2 | N |
| D0N0P5 |  | *P. infestans* | Glycoside hydrolase | 2 | N |
| D0NNP3 |  | *P. infestans* | Glycoside hydrolase | 2 | Y |
| D0NXX6 |  | *P. infestans* | Glycoside hydrolase | 2 | Y |
| G4ZHV1 | D0MV27 (64) | *P. infestans* | Glycoside hydrolase | 3 | N |
| G4ZHV2 | D0MV27 (87) | *P. infestans* | Glycoside hydrolase | 2 | N |
| H3GC44 | D0N0P5 (88) | *P. infestans* | Glycoside hydrolase | 3 | Y |
| H3GJN0 | D0N0P5 (64) | *P. infestans* | Glycoside hydrolase | 4 | N |
| H3GF09 | D0N0P5 (84) | *P. infestans* | Glycoside hydrolase | 3 | Y |
| G4Z4L7 | D0MTV6 (95) | *P. infestans* | Inositol-3-phosphate synthase | 2 | N |
| H3GI93 | D0NCR4 (72) | *P. infestans* | Mucin-like protein | 2 | Y |
| Q3L578 |  | *P. megakarya* | Necrosis and ethylene-inducing protein 1 | 2 | N |
| Q3L570 |  | *P. megakarya* | Necrosis and ethylene-inducing protein 7 | 1 | N |
| G4ZP65 | G4ZA69 (67) | *P. sojae* | Necrosis inducing-like protein NPP1 type | 1 | Y |
| Q8LKL0 |  | *P. sojae* | Necrosis-inducing-like protein | 2 | Y |
| T2FFK2 |  | *P. capsici* | NLP effector | 2 | Y |
| Q0ZJ78 |  | *P. capsici* | PCPG3 | 3 | N |
| G2XKV6 |  | *P. capsici* | Pectate lyase | 1 | Y |
| T1NXE7 |  | *P. capsici* | Pectate lyase | 2 | Y |
| G2XKU9 |  | *P. capsici* | Pectinesterase | 2 | Y |
| G2XKV0 |  | *P. capsici* | Pectinesterase | 2 | Y |
| G2XKV3 |  | *P. capsici* | Pectinesterase | 1 | Y |
| H3GAQ8 |  | *P. ramorum* | Phosphoglycerate kinase | 3 | N |
| T1NXF1 |  | *P. capsici* | Polygalacturonase | 3 | Y |
| T1NXG1 |  | *P. capsici* | Polygalacturonase | 3 | Y |
| H3G816 | G4ZL46 (86) | *P. sojae* | Polygalacturonase | 2 | Y |
| G4ZNE7 |  | *P. sojae* | Putative endo-1,3-beta-glucanase | 3 | Y |
| G4ZEC3 |  | *P. sojae* | Putative glycoside hydrolase family 30 | 4 | Y |
| H3G7U3 | G4YUK6 (100) | *P. sojae* | Putative glycosyl hydrolase family 7 | 2 | N |
| G4YUK7 |  | *P. sojae* | Putative glycosyl hydrolase family 7 protein | 4 | N |
| D0MSJ6 |  | *P. infestans* | Putative uncharacterized protein | 2 | Y |
| D0N018 |  | *P. infestans* | Putative uncharacterized protein | 3 | Y |
| D0N3Y4 |  | *P. infestans* | Putative uncharacterized protein | 2 | Y |
| D0N6G9 |  | *P. infestans* | Putative uncharacterized protein | 5 | Y |
| D0N9N0 |  | *P. infestans* | Putative uncharacterized protein | 5 | Y |
| D0NAQ9 |  | *P. infestans* | Putative uncharacterized protein | 3 | N |
| D0NG32 |  | *P. infestans* | Putative uncharacterized protein | 2 | N |
| D0NG34 |  | *P. infestans* | Putative uncharacterized protein | 3 | Y |
| D0P3Q9 |  | *P. infestans* | Putative uncharacterized protein | 3 | N |
| G4YLH1 |  | *P. sojae* | Putative uncharacterized protein | 2 | Y |
| G4YP82 |  | *P. sojae* | Putative uncharacterized protein | 3 | Y |
| G4YP90 |  | *P. sojae* | Putative uncharacterized protein | 2 | Y |
| G4YS06 |  | *P. sojae* | Putative uncharacterized protein | 2 | Y |
| G4Z433 |  | *P. sojae* | Putative uncharacterized protein | 2 | N |
| G4ZRY1 |  | *P. sojae* | Putative uncharacterized protein | 2 | Y |
| G4ZYR9 |  | *P. sojae* | Putative uncharacterized protein | 3 | Y |
| G5A3Z2 |  | *P. sojae* | Putative uncharacterized protein | 2 | Y |
| G5A5B6 |  | *P. sojae* | Putative uncharacterized protein | 2 | Y |
| G5AB98 |  | *P. sojae* | Putative uncharacterized protein | 3 | Y |
| H3GDN4 | D0NSG4 (65) | *P. infestans* | SCP-like extracellular protein | 1 | Y |
| H3H0J0 | D0N3P2 (71) | *P. parasitica* | Secretory protein OPEL | 4 | Y |
| Q4TUC7 |  | *P. infestans* | Superoxide dismutase | 2 | N |
| G4ZZW1 | B0B0Q5 (81) | *P. cinnamomi* | Transglutaminase elicitor | 4 | Y |
| Q6XDM3 |  | *P. infestans* | Transglutaminase elicitor M81C | 3 | Y |
| H3G7W2 | Q6XDM3 (76) | *P. infestans* | Transglutaminase elicitor M81C | 1 | Y |
| G5A054 | Q6XDM3 (74) | *P. infestans* | Transglutaminase elicitor M81C | 2 | N |
| G4ZZV6 | D0NUH0 (64) | *P. infestans* | Transglutaminase elicitor | 2 | N |
| G4ZZW4 | D0NUH0 (64) | *P. infestans* | Transglutaminase elicitor | 2 | N |
| H3GZF4 | D0NUH0 (70) | *P. infestans* | Transglutaminase elicitor | 2 | Y |
| H3GZF6 | D0NUH0 (60) | *P. infestans* | Transglutaminase elicitor | 2 | Y |
| D0RLV7 |  | *P. infestans* | Transglutaminase elicitor-like protein | 3 | N |
| D0NAC8 | D0NUH1 (84) | *P. infestans* | Transglutaminase elicitor-like protein | 1 | N |
| E5D6U5 |  | *P. megakarya* | Translation elongation factor 1 alpha | 2 | N |
| G4ZB28 | D0P2W7 (95) | *P. infestans* | Triosephosphate isomerase/glyceraldehyde-3P dehydrogenase | 3 | N |
| H3G8V5 |  | *P. ramorum* | Uncharacterized protein | 3 | N |
| H3GCV1 |  | *P. ramorum* | Uncharacterized protein | 2 | Y |
| H3GF08 |  | *P. ramorum* | Uncharacterized protein | 2 | Y |
| H3GFN5 |  | *P. ramorum* | Uncharacterized protein | 2 | Y |
| H3GH67 |  | *P. ramorum* | Uncharacterized protein | 2 | Y |
| H3GK69 |  | *P. ramorum* | Uncharacterized protein | 3 | N |
| H3GPM4 |  | *P. ramorum* | Uncharacterized protein | 2 | Y |
| H3GSM9 |  | *P. ramorum* | Uncharacterized protein | 2 | Y |
| H3H317 |  | *P. ramorum* | Uncharacterized protein | 2 | Y |
| H3H844 |  | *P. ramorum* | Uncharacterized protein | 2 | Y |
| H3H869 |  | *P. ramorum* | Uncharacterized protein | 3 | Y |
| H3HAX6 |  | *P. ramorum* | Uncharacterized protein | 4 | Y |
