# Supplementary material for: Molecular Profiling of the Phytophthora plurivora Secretome: A Step towards Understanding the Cross-Talk between Plant Pathogenic Oomycetes and Their Hosts
Source: PLoS One. 2014 Nov 5;9(11):e112317. doi: 10.1371/journal.pone.0112317 (PMC4221288; doi:10.1371/journal.pone.0112317)
Supplement: Table S3 — List of proteins detected with differential amount in P. plurivora culture filtrates (He) following treatment with the root exudate of Fagus sylvatica (He+RE). The quantification was performed by isobaric labeling coupled to LC-MS/MS analysisa. The secretion prediction according to signal peptide probability of Signal P 4.1 server is reported; Y and N indicate the presence or absence of the signal peptide for secretion. (DOCX) [file pone.0112317.s003.docx]

**Table S3.** List of proteins detected with differential amount in *P. plurivora* culture filtrates (He) following treatment with the root exudate of *Fagus sylvatica* (He+RE). The quantification was performed by isobaric labeling coupled to LC-MS/MS analysis^a^. The secretion prediction according to signal peptide probability of Signal P 4.1 server is reported; Y and N indicate the presence or absence of the signal peptide for secretion.

| **Uniprot AC** | **Blast Match AC (% identity)** | **Species** | **Protein description** | **#Peptides** | **Ratio He/He+RE** | **p-value** | **SignalP** |
| --- | --- | --- | --- | --- | --- | --- | --- |
| G4ZEQ8 | D0MT61 (92) | *P. infestans* | D-isomer specific 2-hydroxyacid dehydrogenase | 2 | 5.4 | 0.0009 | N |
| D0NG32 |  | *P. infestans* | Putative uncharacterized protein | 2 | 4.3 | 0.0079 | N |
| G4YUK7 |  | *P. sojae* | Putative glycosyl hydrolase family 7 protein | 4 | 3.2 | 0.0005 | N |
| D0NXX6 |  | *P. infestans* | Glycoside hydrolase | 2 | 3.2 | 0.007 | Y |
| T2FFK2 |  | *P. capsici* | NLP effector | 2 | 3.1 | 0.0004 | Y |
| H3GZF0 | D0NUH5 (79) | *P. infestans* | Glucan 1,3-beta-glucosidase | 3 | 2.6 | 7E-06 | Y |
| D0MSJ6 |  | *P. infestans* | Putative uncharacterized protein | 2 | 2.6 | 0.0019 | Y |
| D0N0P5 |  | *P. infestans* | Glycoside hydrolase | 2 | 2.5 | 0.0204 | N |
| G5A3Z2 |  | *P. sojae* | Putative uncharacterized protein | 2 | 2.3 | 2E-05 | Y |
| G4ZK12 | G4ZKR2 (69) | *P. sojae* | Avr1b-1 avirulence-like protein | 6 | 2.3 | 6E-09 | Y |
| T1NXE7 |  | *P. capsici* | Pectate lyase | 2 | 2.3 | 0.0013 | Y |
| H3GRB0 | G4ZKR2 (70) | *P. sojae* | Avr1b-1 avirulence-like protein | 2 | 2.2 | 0.0013 | Y |
| G4ZYR9 |  | *P. sojae* | Putative uncharacterized protein | 3 | 2.2 | 0.0159 | Y |
| G4ZHV2 | D0MV27 (87) | *P. infestans* | Glycoside hydrolase | 2 | 2.2 | 0.0036 | N |
| H3GK69 |  | *P. ramorum* | Uncharacterized protein | 3 | 2.0 | 0.0183 | N |
| D0N018 |  | *P. infestans* | Putative uncharacterized protein | 3 | 2.0 | 0.0133 | Y |
| D0NG34 |  | *P. infestans* | Putative uncharacterized protein | 3 | 1.8 | 0.0001 | Y |
| D0RLV7 |  | *P. infestans* | Transglutaminase elicitor-like protein | 3 | 1.6 | 0.0447 | N |
| H3GJN0 | D0N0P5 (64) | *P. infestans* | Glycoside hydrolase | 4 | 1.6 | 3E-12 | N |
| H3GZF6 | D0NUH0 (60) | *P. infestans* | Transglutaminase elicitor | 2 | 1.5 | 0.0056 | Y |
| D0MXT1 |  | *P. infestans* | Endo-1,3(4)-beta-glucanase 1 | 7 | 1.5 | 0.0033 | Y |

^a^ Differential expression was defined by a relative abundance ratio ≥1.5
